# Methods and Lessons From Costing a Large mHealth Intervention at Scale in India

**DOI:** 10.3389/fpubh.2022.894390

**Published:** 2022-06-02

**Authors:** Ritwik Shukla, Avani Kapur

**Affiliations:** Accountability Initiative, Centre for Policy Research, New Delhi, India

**Keywords:** low and middle income countries (LMICs), costing, methods - estimation, planning, budgeting, India, mHealth

## Abstract

The use of mobile devices to deliver public health interventions is rapidly increasing, particularly in low resource settings. Despite their proliferation, several mHealth interventions in developing countries fail to reach geographical scale, and long-term sustainability for most remains uncertain. There is a need to cost for such programs, to enable better planning and budgeting and tailor programs as required. Cost estimates can contribute to a more informed debate on resource allocation priorities and help make choices clearer for policymakers. This paper has two main objectives: (1) present a detailed protocol on determining the costs of a large national mHealth job aid and behavior change communication tool known as Integrated Child Development Services - Common Application Software (ICDS-CAS) in India, and (2) to present lessons for policymakers on how to ensure financial planning for scaling mHealth interventions. The study uses the Activity Based Costing—Ingredients (ABC-I) method. The major advantage of the ABC-I method is the clarity it brings to costs for each input and activity, across levels and geographies. It also accounts for indirect costs. There are five key lessons while costing for mHealth programs. First, that there are many activities and ingredients that must be budgeted for and discussed while planning and implementing mHealth programs. Second, the ABC-I method described in this paper provides great clarity on costs, yet its major limitation is the availability of data, which must be mitigated with the careful use of assumptions. Third, mHealth technology life cycles have financial implications which must be accounted for. Fourth, determining cost locations and all sources of funding including non-government sources is crucial. Fifth, since costing estimates are subject to a set of assumptions, a disaggregation of costs allows for scenario-building, which is useful while planning ahead and accounting for program changes. The evidence generated can be used for more informed debate on resource allocation priorities, given competing priorities in low- and middle-income countries.

## Introduction

The use of mobile and wireless technologies to support the achievement of health objectives or mHealth interventions has been increasing. They have been heralded as having the ability to transform the delivery of health services (1). In particular, mobile phones have the potential to improve access, knowledge, and healthy behaviors. Several studies have also reported that mHealth interventions are cost-effective, economically beneficial, or cost saving (2). Further, mHealth has found a degree of acceptability among health workers who use them as well (3). Given the ever-increasing mobile phone usage in Low and Middle Income Countries (LMICs) (4), the use of mobile devices to deliver public health interventions is believed to be effective, particularly in low resource settings (5).

Despite their proliferation, a systematic review of mHealth interventions found that while the low cost of mobile technology enabled their adoption, successful expansion was often hampered by mechanisms for financial sustainability and a lack of data on the cost of programs at scale (2). Consequently, several mHealth interventions in developing countries fail to reach geographical scale (2), and long-term sustainability for most remains uncertain (6). Sustainable financing is fundamental to the capacity of any mHealth project to increase its scale, yet it is often the most difficult part of the process (7), especially without transferring costs to users (8). Part of the problem when it comes to scaling up, for governments, is the lack of data on the cost of programs at scale (2). Shortage of high-quality data allowing assessments of comparative effectiveness and comparative value makes it difficult for governments to select, scale up, and integrate mHealth solutions into existing national systems (9). While several pilot studies have estimated costs of pilot programs, covering countries such as Nepal (10) as well as states in India such as Uttar Pradesh and Bihar (11, 12), there are only limited studies that have estimated what it would cost to scale-up the program (13).

In the absence of quality administrative data and given financial limitations and several competing priorities in LMICs, economic evaluations are an important tool for effective prioritization of resources (14). Where programs do not have the data, resources, time or expertise to conduct a full economic evaluation, partial evaluations (sometimes referred to as “costing studies”) may be undertaken to measure the costs of a single program (cost description) (15). When done for costs, they can provide cost amounts, key cost drivers, resource estimates required to sustain and scale an intervention, or to develop more comprehensive economic evaluations (16).

This paper attempts to strengthen the evidence base by presenting a comprehensive, bottom-up methodology on undertaking a cost evaluation of a large-scale national mHealth program. We have applied this method to cost for such an intervention in India, known as the Integrated Child Development Services-Common Application Platform (ICDS-CAS). In September 2020, the program was replaced by another application with similar aims known as *Poshan* Tracker (*Poshan* means nutrition).

### Objectives

This study aims to (1) present a detailed protocol on determining the costs of a large national mHealth job aid and behavior change communication tool known as ICDS-CAS in India, and (2) to present lessons for policymakers on how to ensure financial planning for scaling mHealth interventions.

There are two common starting points for any costing study. First, to develop a financial forecast for sustaining and/or expanding program activities; and second, to model the sector-wide implications of scaling up (17). The methods described here solely focus on the former.

### What Was ICDS-CAS?

Launched in 2016, the ICDS-CAS program sought to improve service delivery of nutrition programs and had four key features. First, through a smartphone application, it digitized and automated 10 of the 11 service registers that frontline workers (FLWs) - known as Anganwadi Workers (AWWs) are expected to maintain (except the stock register). This information was then aggregated through a web-based dashboard at different sub-national levels including block, district, state, and finally at the national level. Second, since AWWs are meant to conduct regular home visits to pregnant women and lactating mothers, it had a scheduler that helped prioritize home visits. Third, the software also contained counseling videos which could be used as a job aid. Finally, with features such as a photo capture feature and GPS capability, the application helped create channels for monitoring. AWW supervisors known as Lady Supervisors (LSs) had access to real-time data (18). Between 2016–2019, over 6,00,000 out of 1.4 million community workers had been trained under the program, and INR 640 crore (or 9.1 million USD) had been spent overall as per a Right to Information request filed by the authors.

Studies have found that AWWs spend substantial amounts of time on administrative tasks (19). ICDS-CAS was thus envisaged to reduce this burden. Recent evaluations have also shown that mHealth interventions such as ICDS-CAS can support gains in immediate term service delivery outcomes by enabling more age-appropriate home-visits and counseling but require longer term evaluations to improve other outcomes (20).

Yet, the program was not without its challenges. A process evaluation found that impediments to roll-out included state readiness, delays in device procurement and set-up, dashboard readiness, and low data storage space (21). This study builds on the findings from the process evaluation by focusing on the need for appropriate planning by analyzing costs incurred and required for all components over time.

### Outlining the Protocol

This research uses the Activity Based Costing - Ingredients (ABC-I) method which has been used in several costing studies (22–24). The activity-based costing approach aims to break down the program into a sum of activities. These activities can be described as “cost centers or Activity Based Cost-Centers (AB-CCs) which should be mutually exclusive and exhaustive”. These activities are further broken down into ingredients which are combined to get total costs. The ingredients method requires three pieces of information to derive program costs. (1) list of inputs, (2) the quantities of the inputs used to realize the program, and (3) the cost per unit of an input. In the method described subsequently, these approaches are combined. The output is a detailed cost matrix, which can be used in many ways.

### Possible Applications

Resources are typically scarce in LMICs, and any expenditure at scale requires careful prioritization between competing needs. Cost estimates can contribute to a more informed debate on resource allocation priorities (24), and help make choices clearer for policymakers. Given their disaggregated nature, cost analyses using ABC-I are replicable across programs in other LMICs and can be used by policymakers to plan and budget for new programs, tailor existing programs as they develop, and to determine costs to measure the effectiveness of any program.

The objective of budgeting is estimating existing revenues required and likely expenditures as well as determining future funding needs. As mHealth programs expand across LMICs, every government will have to construct a budget including start-up costs, fixed costs, and variable costs, as well as creating annual or longer-term plans. ABC-I provides a basis for that. Furthermore, since ABC-I aims to disaggregate costs, it can be helpful in creating budgets tailored to a detailed roll-out plan. For example, the program may only be launched in a limited geography or with limited features initially, and gradually expand.

Additionally, as with most government interventions, it is possible that some components of mHealth programs are phased out in the future, or the component-mix and resource-mix requires change. To this end, ABC-I can provide detailed inputs on the minutiae of the program and help visualize these changes easily. For example, this can help in determining the impact of delays or changes in the implementation modalities on total program costs.

Results using the ABC-I method can serve as a toolkit for governments, policymakers, donors, and practitioners. It allows for a comparison with other similar programs that could be rolled out across LMICs in the future.

## Methods

The following five steps were involved in application of the ABC-I approach in this cost evaluation.

### Step 1: Developing a Detailed Description of the Intervention

This process involves sequentially listing out all processes and components that go into implementing the program as desired. This includes, but is not limited to, procurement of equipment, hiring staff, training of FLWs, mid-level managers, and officials, and setting up the monitoring and upkeep procedures. This helps in creating a timeline of all activities conducted for the intervention as well. This description helps in grasping the scope of the intervention and narrowing the focus on activities that require the most time, effort, and resources.

Detailed program descriptions can be created based on operational guidelines set by the implementing authority or be co-created with them. Primary documents used should ideally include the operations manual, impact pathways, training, hiring, and other process manuals, etc. Various partners need to be consulted to understand their exact role, if it is unclear from secondary sources.

### Step 2: Identify and Isolate Activity Based Cost-Centers (AB-CCs)

The procedure for this step is similar to the previous one and some degree of convergence between these steps is anticipated. The detailed checklist created in step one shall be used to identify *mutually exclusive and exhaustive* AB-CCs to avoid double counting and should be sufficiently detailed, to allow for granular analysis of each element of the intervention. A descriptive report helps identify all possible activities, and to break them down by which stage they are needed – start-up, maintenance, or scale-up. Every AB-CC itself comprises various “ingredients” that come together, and the various inputs needed for each activity. For example, training activities could be described as an AB-CC, broken down into ingredients such as FLW training, training of officials and other personnel. Each ingredient requires various inputs such as space to conduct the training, trainer's remuneration, and so on.

### Step 3: List all Ingredients That Go Into an Activity

Every activity must be broken down, as much as possible, into all its elements. This enables the compilation of a cost database, where all inputs and ingredients needed for a given activity are listed in detail. This database specifies the category, ingredient name and description, and a unit of measurement for each (for instance, wages are usually defined as the product of the wage rate per hour).

In this step, vital categories of inputs are delineated—shared vs. non-shared costs, fixed vs. variable costs, and recurrent vs. non-recurrent costs. Capital goods represent a type of fixed and non-recurrent cost which needs to be depreciated over the assets' potential life term. These include vehicles, buildings, etc. Recurrent costs include remuneration for personnel or maintenance costs, which are purchased frequently, often in regular intervals (daily, monthly, or annually). Sometimes for an ingredient, both recurrent and non-recurrent costs exist. For example, while the purchase of a smartphone for the mobile application is a fixed cost it is recurrent every few years because the lifespan of a phone rarely exceeds 2–3 years, whereas the phone servicing and maintenance is a recurrent cost.

This step also entails determining the number of units of each input required for any given activity such as the number of hours of the training agency's time, the number of mobiles and tablets needed, the amount of office space required, and so forth.

The first three steps shall occur simultaneously. Furthermore, they are iterative to an extent whereby new information should be included as and when required.

### Step 4: Compiling Unit Costs

For each activity and ingredient, unit costs are to be compiled from various sources including the government program budget and expenditure documents, training guidelines, procurement registers, etc. There can be certain ingredients for which exact unit costs cannot be compiled. For instance, when personnel work across multiple interventions including mHealth programs, the time they spend and therefore the remuneration paid to them solely for the mHealth program may be unclear. A mitigation strategy for such scenarios is mentioned in the subsection on limitations and mitigation.

Ideally, the cost of any activity should be measured as the total sacrifice made to complete that activity. For this purpose, we may have to use market prices as well as economic costs. It is important to note, however, that market prices may not reflect true economic costs. This is particularly true for inputs that are donated, capital inputs, or have distorted or non-existent markets. For our purpose, the idea is to include time costs, personal costs, and social costs, if any.

To cost for ICDS-CAS, unit costs were obtained from a central Indian state Madhya Pradesh (MP), which was one of the pilot states for the program.

### Accounting for Private and Social Costs

Private costs are those borne by individuals. Health interventions can affect the ability of people to work (they may have to spend more or less time away from work, and therefore affect the total resources available to them. The effect of productivity costs and gains should therefore be included in the study (24). This covers values for non-market items. Some major non-market items include travel costs for various functionaries for the repair, maintenance, replacement of phones, submitting data, charging phones, and so on. Functionaries may not be compensated for certain costs; however, these should be included in the cost profile. Social costs entail costs that are not budgeted for, or don't have markets, and are borne by society or social structures. For most programs, such costs are typically negligible.

### Step 5: Estimating Total Costs

The total cost of each activity or AB-CC can now be calculated, as well as the total cost of the intervention. We have,


$$\begin{array}{l} Total\;Costs\;\left(TC\right)=\;\overset{n}{\underset{1}{\sum }}AB-CC\;Costs \end{array}$$

Where *n* is the total number of AB-CCs.

However, there are several adjustments that must be made when presenting these numbers. First, we need to account for the fact that there is a preference for consumption in the current time period, rather than consumption in any future time period. Therefore, we need to take discounted values for total costs in future years. The WHO recommends a discount rate of 3 per cent, to enable comparisons with other studies (24). In addition, we can use discount rates specific to a country or state as well.

Second, we must index all costs based on inflation rates. That is, we must assess costs in real terms, and not in nominal terms. For example, if the intervention is to be scaled up in 2019, then costs calculated in 2018 must be adjusted for price inflation between 2018 and 2019.

Finally, to account for variability in program design and to test the sensitivity of assumptions, the activity-wise range of costs under different scenarios should be presented.

### Why ABC-I?

The advantages of using the ABC-I method are briefly outlined here. First, ABC-I is a method that provides detailed costs for each input and each activity they go into. Since we put each activity and input under a magnifying lens, calculating marginal costs is easier. Marginal costs are often needed to decide when estimating the scaled-up costs of the program. It is necessary to note that marginal costs are not constant with scale, and ABC-I accounts for that.

Secondly, ABC-I allows for clarity in the reporting of both prices and quantities at various levels and in different contexts. This approach allows us to estimate costs for this program in a different setting (replicability), or on a larger scale (scaling up) without any need to re-collect prices and quantities for different scenarios.

Thirdly, ABC-I accounts for indirect costs that cannot be attributed to a particular program alone. ABC allows us to apportion these costs for each input based on a direct tracing of personnel time to services (25). For example, if personnel work across multiple interventions and only devote part of their time to the mHealth intervention in question.

This methodology is proposed as it brings clarity to the costing exercise by using unit costs, taking into account the possibility of double counting, and by allowing to account for indirect costs (social costs, private costs, among others).

## Results and Discussion

### Key Outputs

The steps listed above lead to the production of two key outputs. First, the construction of a cost profile. This profile lists out the various costs associated with each activity in a comprehensive and disaggregated manner. A cost profile can list activities based on whether they are start-up costs, maintenance costs, or costs sustained during the scale-up process. We can then visualize the shape of the intervention with greater clarity, with information on which activities and inputs cost the most, require the bulk of maintenance costs, or are the costliest aspects of scaling up. This allows comparisons across activities and can inform discussions on optimizing the intervention.

Second, visualizing scale-up costs. Several mHealth interventions start as pilots but aim to scale-up. While an intervention is scaled up, input quantities, and therefore, costs may not increase in a linear fashion. This is particularly true of fixed costs, such as the physical infrastructure and overhead costs. Excluding certain inputs and costs is relatively straightforward using the ABC-I methodology proposed for this exercise. Simultaneously, it allows us to analyze the costs required to replicate the program in vastly different contexts.

There were several lessons from the application of ABC-I to understand ICDS-CAS costs. They are presented below:

### Lesson 1: Each Activity of mHealth Programs Have Several Ingredients Which Need to Be Budgeted for

**Figure 1:**
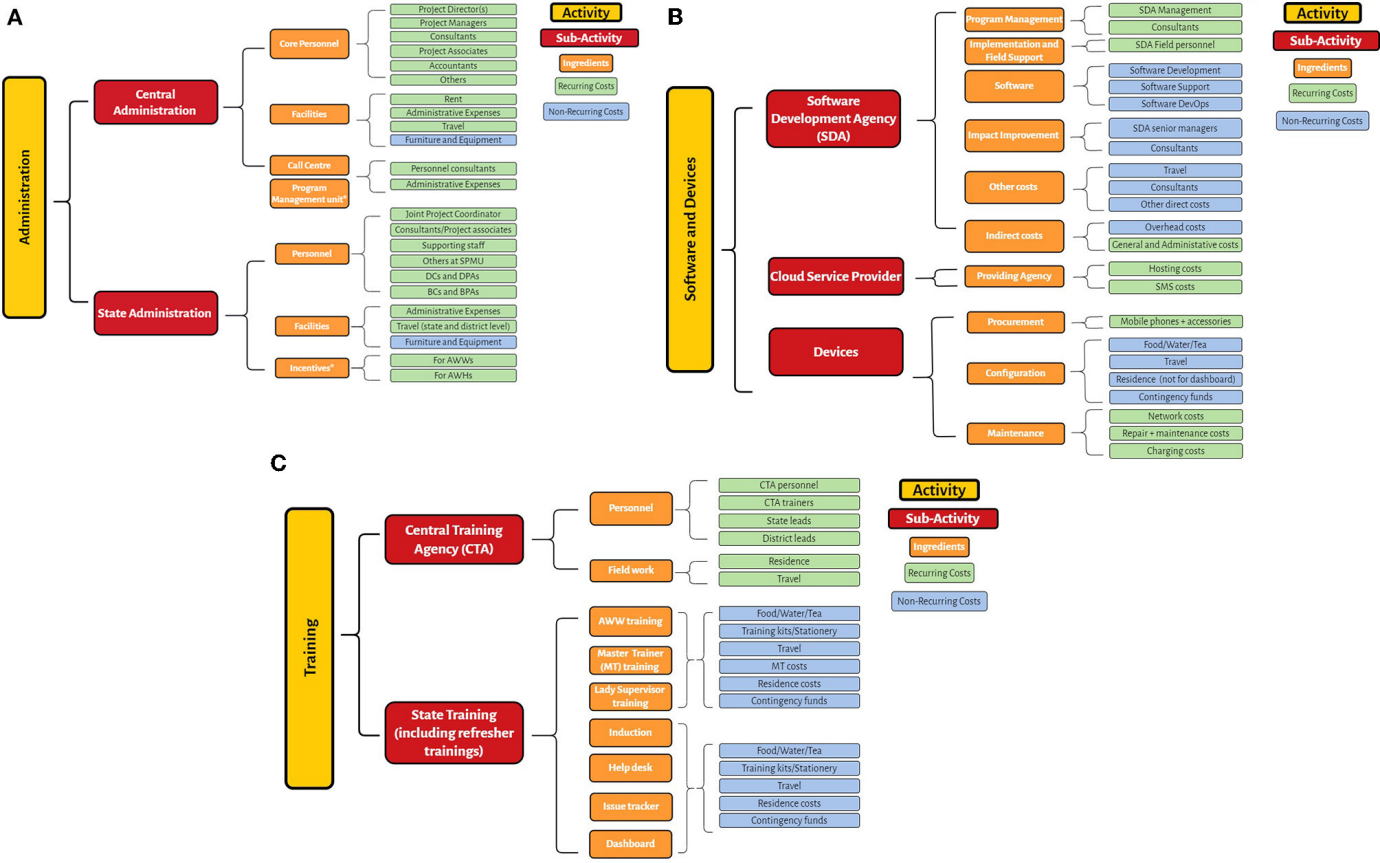
**(A)** Activities and ingredients for ICDS-CAS: Administration. **(B)** Activities and ingredients for ICDS-CAS: Software and Devices. **(C)** Activities and ingredients for ICDS-CAS: Training.

An application of the method to costing for ICDS-CAS, found that broadly, the intervention could be categorized into 3 broad activities (Figure 1):

1. Administrative costs which include all costs related to managing and organizing the implementation of ICDS-CAS. At both the central and state level, project management units were set up to manage CAS, coordinating CAS implementation across states and districts, known as the Central Program Management Unit (CPMU) and the State Program Management Unit (SPMU). These include both non-recurring costs such as facility costs (furniture and equipment) and recurring costs such as personnel costs, expenses on electricity, stationery, etc. Examples of personnel included District Coordinators (DCs), District Project Assistants (DPAs), Block Coordinators (BCs), Block Project Assistants (BPAs)
2. Software and devices including costs for the software developer, Cloud Service Provider (CSP), and devices. Data collected through mHealth interventions requires huge cloud storage capacity. Simultaneously, device costs include not just the purchase of devices and accessories, but also costs to maintain and repair devices, and monthly usage fees.
  The software component includes all costs incurred on developing the CAS platform incurred primarily by the Software Development Agency (SDA). The ingredients for the software developer include program management, software development, data analytics, impact assessment and improvement, field support, and travel.
3. Training costs include costs for the Training Agency (TA), and related training costs. The two main parts to training costs are designing and implementing the training, and providing the resources (location, food, etc.) for the same.

Each of the major components (administrative costs, software and device costs, and training costs in the case of ICDS-CAS) have several ingredients which need to be budgeted for. Moreover, programs must be augmented with continuous training, supervision and the provision of equipment as well as planning costs over time (26). An example of the cost profile for ICDS-CAS is given in the format below (Table 1).

**Table 1:** An example of assumptions while costing for ICDS-CAS.

**Type**	**Assumption and method**
Personnel time costs	Costs for setting up and managing the CPMU are taken from guidelines issued on 26.02.18. Since these costs are for the scheme as a whole and not just for the specific mHealth intervention, we have apportioned 70% of the total CPMU costs to CAS in year 1, 50% in year 2, and 30% in year 3 and thereafter.
	Since states oversee implementation, we assumed that 90% of time of SPMU resources in year 1, 70% in year 2, and 50% in year 3 and thereafter (personnel and office costs).
Absence of Cloud Storage Provider Costs	We calculated average month wise per AWW costs and multiplied that by the number of AWWs in a state. This assumes that cloud storage provider costs scale linearly.
Software Developer	For calculating scale-up costs, centrally incurred costs of software are based on total grant given for software development. Further, additional administrative costs of the software developer were also included.
Life span related assumptions for Devices	We assumed a device has an average life of 3 years.

### Lesson 2: The Major Limitation of ABC-I Is Data Availability, but It Can Be Mitigated

There are many kinds of data gaps that may exist. First, certain costs may be unavailable. With government programs, several costs might be sensitive, and access may be restricted. It is also possible that figures obtained may be estimates themselves. For instance, while applying the ABC-I method to cost for ICDS-CAS in India, detailed data on cloud storage costs, costs for maintaining the call center, and costs for the program management unit were not available and had to be inputted based on limited information. For pilot programs, it is also hard to predict how large or small certain costs would be if the program scaled up. The lack of cost data has been a challenge while evaluating other mHealth interventions as well (27).

Second, estimating exact annual costs is difficult, given the dynamic evolution of programs. Several programs are rolled out in a phased manner, starting with a pilot. In the absence of information on how the program would evolve in the next, say 5–10 years, costing must be based on current guidelines. Therefore, changes in costing due to economies of scale, technological dividends, or a change in the implementation design if the scale-up is staggered are hard to account for.

Relatedly, as programs are scaled up, costs across geographical settings might not be available in disaggregated form. In a country like India, for instance, every state may eventually have a slightly different implementation model based on their current technological and administrative capacity. This would impact how the intervention is scaled and costed. Finally, assigning costs for personnel working across interventions and programs might be challenging in absence of a detailed time-use study, as mentioned before.

To mitigate some of these limitations, assumptions can play a critical role. These could be assuming proxy values for certain costs, assuming time spent by personnel on the program, more general things such as identifying different sources, and setting discount rates, inflation rates, conversion rates, etc. All these assumptions must be carefully listed out to enable replication and justified as well.

The sensitivity of each assumption should also be tested i.e., how much do costs change with changing assumptions. For instance, how much do total costs vary if we assume that certain personnel spend 50% of their time on the mHealth program instead vs. if this figure were 70%. These assumptions are useful in calculating the range of possible costs under different sets of assumptions and should be used as a guide to a more comprehensive planning and budgeting exercise. An example of some assumptions, used when calculating costs for ICDS-CAS are given below.

A related strategy is to leverage data collected by various studies and surveys which analyze different aspects of programming including time use of personnel. This data can be directly used while inputting unit costs, or indirectly in the form of using it in assumptions to proxy for certain costs.

### Lesson 3: mHealth Technology Life Cycles Have Financial Implications

Overall costs of the program are the most important costs to highlight, as well as per FLW costs. Along with this, costs for each component should be noted as well. For example, for ICDS-CAS, 55% of total costs at scale would have been on buying devices. This is then followed by the cost of conducting state level training of AWWs and LSs, at 18%, and state administration at 17% of the total costs incurred.

Any cost description should differentiate between recurring fixed costs, variable costs, and onetime capital costs. As an example, within the AB-CC ‘software and devices', cloud storage costs are all recurring costs, while all costs for the software developer are non-recurring.

Overall, for ICDS-CAS, in year 1, non-recurring costs account for 59% of total costs with the bulk being costs for devices (75% of recurring costs) and training (15% of recurring costs) (Table 2). Their proportions however decreased significantly in subsequent years. Instead, the proportion of recurring costs related to administrative costs and cloud storage increased. Accounting for these changes is useful to plan resources, particularly for the medium term.

**Table 2:** Ratio of Recurring and non-recurring costs across activities.

**Activity**	**Ratio of recurring costs**	**Ratio of Non-recurring costs**
Central Administration	86	14
State Administration	94	6
**Administration**	94	6
Software Developer	0	100
Cloud service provider	100	0
Devices	20	80
**Software and Devices**	25	75
Training Agency	0	100
State training	50	50
Training	45	55
Total	41	59

At the same time, for financial sustainability of mHealth interventions, it is important to remember the life cycle of technology. The cyclical nature of fixed costs must therefore be accounted for over time. Over time, costs across programs can also be reduced by avoiding monolithic architecture and investing in interoperability (6).

**Figure 2:**
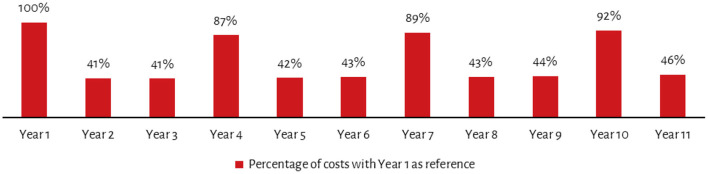
Year-wise costs as a proportion of year 1 costs.

Certain non-recurring costs such as devices and other equipment may need to be replaced every 3–5 years. Extending this analysis over a 10-year period for ICDS-CAS and accounting for inflation and discount future spending in present time, shows a non-linear cost progression. Thus, while year 4, 7 and 10 sees significant spikes in costs due to replacement of devices and other equipment, for the remaining years costs are less than half the costs incurred in year 1 (Figure 2). Moreover, following year 1, costs would be highest in year 10 which in addition to replacement of devices, assumes replacement of other furniture and equipment. Similarly, since it is assumed that most training will be conducted in year 1, the training costs in subsequent years pertain only to refresher training and thus decrease from the second year.

### Lesson 4: Determine Cost Locations and all Sources of Funding Including Non-government Sources

In the early stage of large mHealth interventions, sources of funding may include non-government ones including donor funding, implementing organizations responsible for training etc. It is thus useful to disaggregate costs based on the source of funding for each component. Paired with plans to spend funds over time, this also ensures that the funds are sourced appropriately, and advance planning can ensure the government has funds coming through for the program. Furthermore, it should attempt to quantify any in-kind contributions they may receive from other organizations, and identify cost-share opportunities (7).

Restrictions on the use of government money may impede implementation by impacting procurement, including the procurement of third-party software and hardware and ongoing maintenance and support from vendors with the necessary skill sets. Continued donor funding can be difficult to sustain but nevertheless provides added flexibility to be responsive to evolving needs on the ground (6).

For ICDS-CAS, costs such as central administration and cloud storage were fully funded by the Union government. For the others, costs were shared between the Union government and states in an 80:20 ratio for large states, 95:5 for hilly states, and 100:0 for UTs, as per scheme cost-sharing norms. In India, based on this fund sharing pattern, a majority (78%) of the total scaled up costs for ICDS-CAS in year 1 was to be funded by the Union government. State governments combined would have to fund 17% of the total costs. The remaining costs for training and software development were to be borne by non-government entities.

### Lesson 5: Cost Estimates Are Subject to a Set of Assumptions, Which Allows for Scenario-Building

One of the major challenges of costing studies is the absence of disaggregated financial data (28). To address this challenge, a series of clear assumptions must be used and disclosed to ensure studies can be replicable. Another advantage of using different assumptions is that it allows for scenario building. For example, what would costs be if the program were scaled only to some regions and not the entire country; what would costs be if programmatic norms were different say, regarding training of FLWs; how would costs change with different unit costs; and how would costs vary with the addition of new components. These scenarios provide various paths that the program can take, and can help decision-makers make more informed choices. Some examples are given below, in the context of ICDS-CAS (Table 3).

**Table 3:** Cost variability as a proportion of expected costs due to changes in assumptions: an example.

	**Minimum cost scenario**	**Expected/presented scenario**	**Maximum cost scenario**
**Central Administration**			
Assumptions	30% personnel time spent on CAS; All equipment and furniture available; No project management unit (PMU)	70% personnel time spent on CAS; Equipment and furniture purchased as per national guidelines; No project management unit	100% personnel time spent on CAS; Equipment and furniture purchased as per national guidelines; PMU costing information as per key stakeholder(s)
Amount as a proportion of expected costs	42%	100%	761%
**State Administration**			
Assumptions	50% personnel time spent on CAS, Costs from the pilot state (MP); All equipment and furniture available; No incentives given	90% personnel time spent on CAS, Costs from the pilot state (MP); Equipment and furniture purchased as per MP norms; No incentives given	100% time spent on CAS, Costs as per national guidelines; All equipment and furniture available; Incentives given and all FLWs meet inclusion criteria
Amount as a proportion of expected costs	90%	100%	569%
**Software and Devices**			
Assumptions	Costs for devices, network, and configuration based actual expenditure in the pilot state (MP); No additional support by TA	Costs for devices, network, and configuration based actual expenditure in the pilot state (MP); No additional support by TA	Device and network costs as per national guidelines; Configuration costs as per actual expenditure in the pilot state (MP); Additional support costs as per TA; additional SDA costs incurred
Amount as a proportion of expected costs	100%	100%	194%
**Training**			
Assumptions	Initial training: based on norms in the pilot state and costs as per national guidelines; Refresher training: Norms and costs as per national guidelines, non-residential training; No additional TA support	Initial training: based on norms in the pilot state and costs as per national guidelines; Refresher training: based on norms in the pilot state and costs as per national guidelines, residential training for district and block officers; No additional TA support	Initial training: based on norms in the pilot state, residential training for FLWs; Refresher training: Norms and costs as per national guidelines, residential training for all; Continued TA support with 1 person per for 6 months
Amount as a proportion of expected costs	95%	100%	287%
**Overall costs**			
Total	97%	100%	265%

In the absence of information on exact time spent by the CPMU and SPMU staff on ICDS-CAS specifically, assumptions had to be made regarding personnel time, as mentioned above. Administrative costs account for only 17% of total costs and thus changing the assumption did not significantly impact the overall costs.

On the other hand, software and device costs are driven primarily by device costs and the support required for configuration. The purchase price for devices can thus alter costs in a significant way. To the extent that the application doesn't change radically in the future, we can assume that device costs should not rise in the future. In fact, devices have been getting cheaper over time. However, if the application requires a better device for optimal performance, costs may rise. Therefore, total program costs are sensitive to the way the application will be structured in the future, and the way the application is used.

For training, costs vary substantially based on the amount of support to be provided by the TA, whether the training is to be residential or not, and whether some groups require refresher training. For ICDS-CAS, the main assumption impacting training costs is whether training is residential or not.

## Conclusion

Mhealth interventions for health and nutrition have been studied at length. There are three primary gaps which have been highlighted which are addressed with the methods and lessons described above. First, is the reporting on program sustainability, scale-up costs, and long-term effects of the intervention (29). Second, all the active ingredients of the intervention are reported in sufficient detail (30). Relatedly, these components should be reassessed before scaling, with an emphasis on effectively responding to government procurement and distribution challenges, which should be costed for. Third, sustainability strategies tend to focus on initial capital investment rather than ongoing recurring costs (31).

Adding to the literature, the study presents the method from the first attempt at costing a large-scale national mHealth intervention in India. In India's context, applicable to other developing countries as well, there are several crucial related lessons related to scaling up. The study highlights five key lessons while costing for mHealth programs. First, that there are many activities and ingredients that must be budgeted for and discussed while planning and implementing mHealth programs. Second, the ABC-I method described in this paper provides great clarity on costs, yet its major limitation is the availability of data. Given that ABC-I aims to be as comprehensive and disaggregated as possible, a large amount of data is required. The lack of data for activities and inputs must be mitigated with the careful use of assumptions. These assumptions should be clearly listed out, and their sensitivity should be tested as well, as an indication of robustness of the costing study. Third, mHealth technology life cycles have financial implications. This includes understanding the life cycle of the technology used. For example, phones must be replaced every 2–3 years, which must be budgeted for. Fourth, in the early stages of a large mHealth intervention, sources of funding may include non-government sources. Embedding digital health solutions into health systems will require transitioning management and ownership to government partners who will have to bear the additional costs. Fifth, since costing estimates are subject to a set of assumptions, a disaggregation of costs allows for scenario-building, which is useful while planning ahead. Pilot costs are very different from costs at scale, and in fact, the component mix may be completely different, which should be kept in mind. As they say, you may pilot “apples” but have to scale “oranges” (31). At the same time, defining governance structures and roadmaps up front is helpful while scaling and taking decisions over future program pathways (31).

As mHealth programs expand globally, governments and policymakers require a lot of information not just on their effectiveness, but on financial implications as well. For full-scale sustainability, financing for all aspects of digital health solutions needs to be integrated into routine health budgets and the budgeting processes. Gathering and estimating program costs is essential for LMICs, given that resources are typically scarce and there are several competing priorities. Project teams should also keep in mind that drafting a budget should not be a one-off event. Budgets should be revisited regularly since funding, assumptions and activities can all change. Regular reviews will assist project teams to plan appropriately for increasing their scale and managing resources efficiently (7). This entails not just costing and budgeting for the long term. Given that building consensus around the roles of national and state governments, implementing and technical partners and donors has been a challenge, it entails sustaining relationships that navigate the corridors of power between different stakeholders across governments and funding agencies (6, 31).

Exercises such as the costing method described above can be used by policymakers to plan and budget for new programs, tailor existing programs as they develop, and to determine the cost-effectiveness of any program. For India specifically, the government of India has replaced ICDS-CAS with a new mHealth intervention known as *Poshan* Tracker, and similar interventions are being launched globally. With that in mind, we hope that this analysis can help assess the resources necessary to undertake or sustain similar interventions including in low-resource settings.

## Data Availability Statement

The original contributions presented in the study are included in the article/supplementary material, further inquiries can be directed to the corresponding author.

## Author Contributions

AK and RS conceptualized the paper outline. RS conducted the literature review with inputs from AK. RS collected and analyzed the data, while AK reviewed the data analysis. RS wrote the first draft of the manuscript and AK reviewed it and provided feedback, suggestions, and comments. AK also edited significant sections of the manuscript. Both authors contributed to manuscript revision, read, and approved the submitted version.

## Funding

This study was funded by Grant No. OPP1158231 from the Bill and Melinda Gates Foundation (BMGF) to the University of California, San Francisco.

## Conflict of Interest

The authors declare that the research was conducted in the absence of any commercial or financial relationships that could be construed as a potential conflict of interest.

## Publisher's Note

All claims expressed in this article are solely those of the authors and do not necessarily represent those of their affiliated organizations, or those of the publisher, the editors and the reviewers. Any product that may be evaluated in this article, or claim that may be made by its manufacturer, is not guaranteed or endorsed by the publisher.
